# Integrated Analysis of Structural Variation and RNA Expression of FGFR2 and Its Splicing Modulator ESRP1 Highlight the *ESRP1*^amp^-*FGFR2*^norm^-*FGFR2-IIIc*^high^ Axis in Diffuse Gastric Cancer

**DOI:** 10.3390/cancers12010070

**Published:** 2019-12-25

**Authors:** Sara Pinto Teles, Patrícia Oliveira, Marta Ferreira, Joana Carvalho, Pedro Ferreira, Carla Oliveira

**Affiliations:** 1 Ipatimup—Institute of Molecular Pathology and Immunology, University of Porto, Rua Júlio Amaral de Carvalho 45, 4200-135 Porto, Portugal; 2 i3S—Instituto de Investigação e Inovação em Saúde, University of Porto, Rua Alfredo Allen 208, 4200-135 Porto, Portugal; 3 Master in Oncology, Institute of Biomedical Sciences Abel Salazar, University of Porto (ICBAS-UP), 4050-313 Porto, Portugal; 4 Department of Computer Science, Faculty of Sciences, University of Porto, Rua Campo Alegre 1021/1055, 4169-007 Porto, Portugal; 5 Department Pathology and Oncology Faculty of Medicine University of Porto, Alameda Prof. Hernâni Monteiro, 4200-319 Porto, Portugal

**Keywords:** gastric cancer, FGFR2, FGFR2-IIIb, FGFR2-IIIc, ESRP1, diffuse

## Abstract

Gastric Cancer (GC) is one of the most common and deadliest types of cancer in the world. To improve GC prognosis, increasing efforts are being made to develop new targeted therapies. Although *FGFR2* genetic amplification and protein overexpression in GC have been targeted in clinical trials, so far no improvement in patient overall survival has been found. To address this issue, we studied genetic and epigenetic events affecting *FGFR2* and its splicing regulator *ESRP1* in GC that could be used as new therapeutic targets or predictive biomarkers. We performed copy number variation (CNV), DNA methylation, and RNA expression analyses of *FGFR2*/*ESRP1* across several cohorts. We discovered that both genes were frequently amplified and demethylated in GC, resulting in increased *ESRP1* expression and of a specific *FGFR2* isoform: *FGFR2-IIIb*. We also showed that *ESRP1* amplification in GC correlated with a significant decreased expression of *FGFR2-IIIc*, an alternative *FGFR2* splicing isoform. Furthermore, when we performed a survival analysis, we observed that patients harboring diffuse-type tumors with low *FGFR2-IIIc* expression revealed a better overall survival than patients with *FGFR2-IIIc* high-expressing diffuse tumors. Our results encourage further studies on the role of *ESRP1* in GC and support *FGFR2-IIIc* as a relevant biomarker in GC.

## 1. Introduction

Gastric Cancer (GC) remains one of the most common and deadliest types of cancer in the world [1]. Although GC incidence and mortality has decreased throughout the years and novel therapies have been developed, less than one fifth of advanced GC patients survive 5 years post disease diagnosis [2,3]. Late diagnosis and high intra/inter-tumor heterogeneity likely explain this dismal prognosis and therapeutic failure [4]. Given the non-curative nature of gastric surgery in patients with advanced cancer, two targeted therapies have been approved to treat these patients: the monoclonal antibodies *Trastuzumab* (anti-HER2) and *Ramucirumab* (anti-VEGFR2) [5,6,7]. 

*Trastuzumab* combined with chemotherapy is given to patients harboring HER2 overexpressing tumors, used as a predictive marker of therapy response, and extends median overall survival in 2.7 months, compared to chemotherapy alone [5] *Ramucirumab* is provided to GC unselected patients, extending their median overall survival in 2.2 months in comparison to conventional chemotherapy [7]. Many other therapies have been tested targeting multiple cancer-associated receptors/ligands but failed to provide any survival benefit [8,9,10,11,12]. Most of these therapies were tested without resourcing to predictive markers of therapeutic response, and this may justify their inefficiency. Therefore, understanding the molecular complexity of GC to identify valuable predictors of therapy response is urgent to decrease/delay mortality in this disease.

Antibodies targeting FGFRs, a known family of receptors often dysregulated in cancer, have been used in several GC clinical trials [13,14]. Given the reported FGFR2 amplification/overexpression in GC, FGFR2 signaling has been for long considered a good candidate for new targeted therapies in this disease [15,16,17,18]. For example, Su et al. [17] reported 7.4% of *FGFR2* amplification in a UK GC cohort, while TCGA consortium [18] described a maximum of 9% for specific GC molecular subtypes. Nagatsuma et al. reported that 31.1% of GCs presented FGFR2 protein overexpression, while Tokunaga et al. extended this observation to 61% in a cohort of esophagogastric junction adenocarcinoma [15,19]. These and other studies triggered several clinical trials using different FGFR2-targeting antibodies in unselected GC patients, but with no survival benefit [12,20] (e.g., clinical trial #NCT01719549). The fact that *FGFR2* locus encodes two main isoforms with distinct expression patterns (the epithelial-specific FGFR2-IIIb and the mesenchymal FGFR2-IIIc isoforms), may contribute to this failure [21,22]. The difference between these two isoforms lies on their third immunoglobulin domain, which leads to different binding affinities to FGFR ligands and distinct activation of downstream signaling pathways [21,23,24]. 

In cancer, FGFR2 isoform dysregulation has been widely observed. FGFR2-IIIb overexpression has been detected in cervical, esophageal and pancreatic cancer [25,26,27]. Particularly in pancreatic, but also in lung cancer, expression of FGFR2-IIIb and its main ligand FGF7, have been associated with poor prognosis [28,29]. In contrast, FGFR2 down-regulation has been reported in bladder, prostate and salivary gland cancer [30,31,32,33]. Interestingly, induced overexpression of FGFR2-IIIb in salivary gland, malignant prostate and bladder cancer cell lines led to decreased cell and tumor growth [33,34,35]. Altogether, these studies revealed that FGFR2-IIIb isoform may have both oncogenic and tumor-suppressive effects in a tissue-dependent manner. Regarding FGFR2-IIIc, its expression has been thoroughly studied in the context of Epithelial-to-Mesenchymal Transition (EMT). *FGFR2-IIIb* is the major isoform in epithelial cells, while *FGFR2-IIIc* isoform becomes overexpressed when cells transit to a mesenchymal state [36,37]. In cancer, this switch appears to be rare, nevertheless it has been observed during prostate cancer progression and from normal kidney to clear cell renal cell carcinoma (ccRCC) [38,39]. Furthermore, in ccRCC, *FGFR2-IIIc* expression was found to be correlated with higher tumor grade and worse prognosis [39]. In GC, different studies have reported FGFR2-IIIb overexpression in up to 4% of analyzed cases, most of which presenting *FGFR2* genetic amplification [40,41]. Of notice, Han et al. showed a strong association between FGFR2-IIIb RNA and protein expression in a large GC cohort [41]. Currently, there is one clinical trial testing the efficacy of an anti-FGFR2-IIIb antibody (*Bemarituzumab*) in combination with FOLFOX6 in GC (clinical trial #NCT03694522). In this study, patients are being selected based on FGFR2-IIIb protein overexpression or *FGFR2* genetic amplification. Encouragingly, in a preliminary dose-finding study with this antibody, 4/21 patients with FGFR2-IIIb overexpression (gene amplification or protein overexpression) presented partial response to treatment [42] (clinical trial #NCT02318329). This shows that other mechanisms triggering aberrant *FGFR2* isoform expression in GC, may also be relevant for patient stratification. For example, Park et al. showed that *FGFR2* promoter methylation status was correlated with *FGFR2* RNA expression in a panel of GC cell lines [43]; however this association was never assessed in actual patients’ neoplastic material. Although studies reported FGFR2-IIIb as the most represented isoform in *FGFR2*-amplified GC cases, the frequency of FGFR2-IIIc expression has not been assessed.

ESRP1 is the main regulator of *FGFR2* alternative splicing and promotes splicing of *FGFR2-IIIb* in epithelial cells in detriment of FGFR2-IIIc. During EMT, as epithelial cells transdifferentiate into mesenchymal cells, ESRP1 and FGFR2-IIIb expression decreases, while FGFR2-IIIc increases [44,45]. The role of ESRP1 in FGFR2 isoform expression was never addressed in GC and data on *ESRP1* (epi)genetic status is also scarce. Nevertheless, it has been shown that 50% of a large GC cohort presented copy number gain across the region encompassing the *ESRP1 locus* (8q22, [46]). Although consequences of ESRP1-induced alternative splicing have been explored across several cancer types, showing both an oncogenic and tumor-suppressing effect, it has yet to be verified in GC [47,48,49].

The overall aim of this study was to explore genetic and epigenetic events affecting the expression of *FGFR2* isoforms and their splicing regulator *ESRP1*, as well as their correlation and potential clinical impact. This knowledge is expected to shed light into better predictive markers of response to anti-FGFR therapy in GC.

## 2. Results

Herein, we explore the (epi)genetic regulation and expression pattern of FGFR2, its isoforms and splicing regulator ESRP1, in normal and tumor stomach samples, and potential associations with clinico-pathological and survival data.

### 2.1. FGFR2 and ESRP1 Are Frequently Amplified and Exhibit Promoter Demethylation in GC

**Figure 1:**
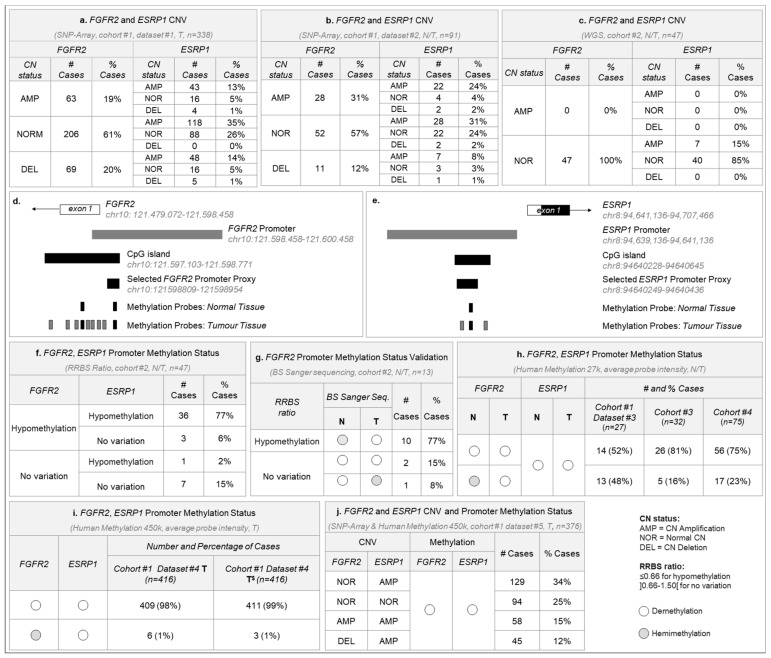
*FGFR2* and *ESRP1* somatic copy number and promoter methylation status across several gastric cancer cohorts. (**a**) *FGFR2* and *ESRP1* Copy number status for cohort #1 dataset #1 tumor samples (TCGA, *n* = 338 GC unpaired samples). Most samples display amplification for at least one of the genes. (**b**) Same as (a) for cohort #1 dataset #2 tumor samples (TCGA, *n* = 91 normal and GC paired samples). Most samples display amplification for at least one of the genes. (**c**) Same as (**a**) for cohort #2 tumor samples (*n* = 47 normal and GC paired samples). Most samples present normal copy number for both genes. (**d**) Representation of the 5’ region of the *FGFR2* human *locus*, the promoter analyzed by Reduced Representation Bisulfite Sequencing (RRBS) in cohort #1, the predicted CpG island, the region selected for Bisulfite Sanger Sequencing validation (proxy) and the analyzed 9 methylation probes available in normal mucosa and tumor tissue from collected TCGA and GEO datasets. Black probes are those for which information is available for both normal and tumor tissue. (**e**) Representation of the 5’ region of the *ESRP1* human *locus*, the promoter analyzed by RRBS, the predicted CpG island, the region selected as proxy and the analyzed methylation probes available in normal mucosa and tumor tissue from TCGA and GSE datasets. The black probe is the only for which information is available for both normal and tumor tissue. (**f**) *FGFR2* and *ESRP1* promoter methylation status according to the RRBS results for cohort #2. Represented is the ratio: number of CpG sites methylated in the tumor sample divided by the number of CpG sites methylated in the paired normal sample. GC cases with ratios equal or below 0.66 are considered hypomethylated while GC cases with ratios between 0.66 and 1.5 are considered without any variation. No GC cases with ratios above 1.5 (hypermethylated) were identified. (**g**) Results of the Bisulfite Sanger sequencing validation of 13 GC cases selected from within cohort #2. Grey circles correspond to hemimethylated samples while white circles correspond to demethylated samples, determined by the analysis of the corresponding electropherograms. Also represented are the observed RRBS ratios. (**h**) Beta-values calculated for the only probe with data available for cases with paired normal and tumor samples from cohort #1, dataset #3 (TCGA, *n* = 27 cases), cohort #3 (GSE25869, *n* = 32 cases) and cohort #4 (GSE30601, *n* = 75 cases). Only the scenarios with most cases are represented. (**i**) Average beta-value calculated for the 2 probes with representation both in the normal and tumor paired samples from cohort #1 dataset #3, cohort #3 and #4 (*n* = 134 cases), as well as for cohort #1, dataset #4, which encompasses 416 tumor samples (TCGA). For this dataset, it is represented both the average beta-values for analyzed probes in all other cohorts (T), as well as the average beta-value calculated for all available probes overlapping the predicted CpG islands (T$): 9 probes for *FGFR2* and 3 probes for *ESRP1*. Samples are separated per cohort and type: normal (N) or tumor (T). Only the scenarios with most cases are represented. (**j**) *FGFR2* and *ESRP1* CNV and promoter methylation status for cohort #1 dataset #5. Only the scenarios with most cases are represented.

*FGFR2* genetic amplification was observed in 19% (63/338) of stomach tumor samples from TCGA (cohort #1: dataset #1). When tumor was compared directly to its normal counterpart (T*vs*N) (cohort #1: dataset #2), the frequency raised to 31% (28/91) (Figure 1a,b). However, no amplification was observed in T*vs*N from our own cohort #2 cases (Figure 1c). *ESRP1* amplification occurred in over 60% (209/338 and 57/91) of stomach tumors from cohort #1, and in 15% (7/47) of cohort #2 tumors (Figure 1a–c). *FGFR2* and *ESRP1* were co-amplified in up to 24% of TCGA tumors when tumor and normal samples were compared. The most frequent combination of events was *FGFR2* normal copy number (CN) and *ESRP1* amplification, observed in 31–35% of TCGA tumors and in 15% of cohort #2 (Figure 1a–c).

Promoter methylation analysis of the region 2000bp upstream of the TSS of *FGFR2* and *ESRP1* revealed that most tumor samples from cohort #1 (datasets #3 and #4), cohorts #2, #3 and #4 were hypo/demethylated for both gene promoters (Figure 1f,h,i). This result was validated by Bisulfite Sanger sequencing for the *FGFR2* promoter in a selected subset of cases from cohort #2 (Figure 1g and Figure S3a). 

To understand the frequency of tumors with the highest potential for a transcriptionally permissive state, we analyzed cohort #1 dataset #5 and observed that 62% (232/376) of the tumors presented concomitantly demethylated *FGFR2* and *ESRP1* promoters and *ESRP1* amplification, with *FGFR2* locus presenting either normal CN (34%, 129/376), amplification (15%, 58/376) or deletion (12%, 45/376) (Figure 1j). Furthermore, 4/4 GC cell lines tested presented fully demethylated *FGFR2* and *ESRP1* promoters (Figure S3b).

### 2.2. FGFR2 and ESRP1 Promoter Demethylation and Amplification Are Correlated with High RNA Expression in GC

Our analysis revealed that most gastric tumors presented hypo/demethylation of both *FGFR2* and *ESRP1* promoters. To elucidate whether *ESRP1* and *FGFR2* demethylation in tumors was associated with higher gene expression, we analyzed available RNA data from cohorts #1 and #2. Overall, total *FGFR2* expression in normal tissue was not different from that in tumors (Figure S4a). Nevertheless, those presenting *FGFR2* promoter demethylation displayed higher RNA expression than the few tumors presenting any degree of methylation (cohort #1 dataset #10, Figure S4b). Unlike total *FGFR2*, *ESRP1* is overall overexpressed in tumors when compared to normal samples (cohorts #1 datasets #6 and #7, *p*-value ranging from 8.93 × 10^−6^ to 9.46 × 10^−3^, Figure S4c). Moreover, TCGA tumors with *ESRP1* promoter demethylation (cohort #1 dataset #10) presented higher RNA expression than the few cases presenting any degree of promoter methylation (Figure S4d). 

To understand whether *FGFR2* and *ESRP1* overexpression was associated with increased CN, we analyzed T*vs*N and unpaired tumor samples from cohort #1 dataset #8 and #9. Although we could not find an association between total *FGFR2* RNA expression and *FGFR2* CN status when comparing T*vs*N, (Figure S4e), we found that tumors with amplified *FGFR2* presented the highest total *FGFR2* RNA expression when compared to tumors bearing normal or deleted *FGFR2* CN (dataset #9, Figure S4f, *p*-value ranging from 1.97 × 10^−7^ to 9.46 × 10^−4^). Tumors with *ESRP1* amplification expressed significantly more *ESRP1* when compared to paired normal samples (*p*-value = 7.93 × 10^−3^, Figure S4g) as opposed to those without amplification. In concordance, tumors with normal or deleted *ESRP1* CN presented lower RNA expression than those with *ESRP1* amplification (*p*-value = 2.2 × 10^−16^ and 1.57 × 10^−6^, Figure S4h). The increase of *ESRP1* expression in T*vs*N derived particularly from tumors presenting, besides amplified *ESRP1* locus, normal *FGFR2* CN (*p*-value = 1.75 × 10^−2^, Figure S4i). Interestingly, when analyzing exclusively tumor data, *ESRP1* expression was significantly increased when the *FGFR2* locus was concomitantly deleted (Figure S4j). Overall, amplification, and most likely promoter demethylation also, of *FGFR2* and *ESRP1* genes correlate well with higher expression levels of both genes in gastric cancers. 

### 2.3. ESRP1 and FGFR2-IIIb Are Overexpressed While FGFR2-IIIc Is Down-Regulated in GC

**Figure 2:**
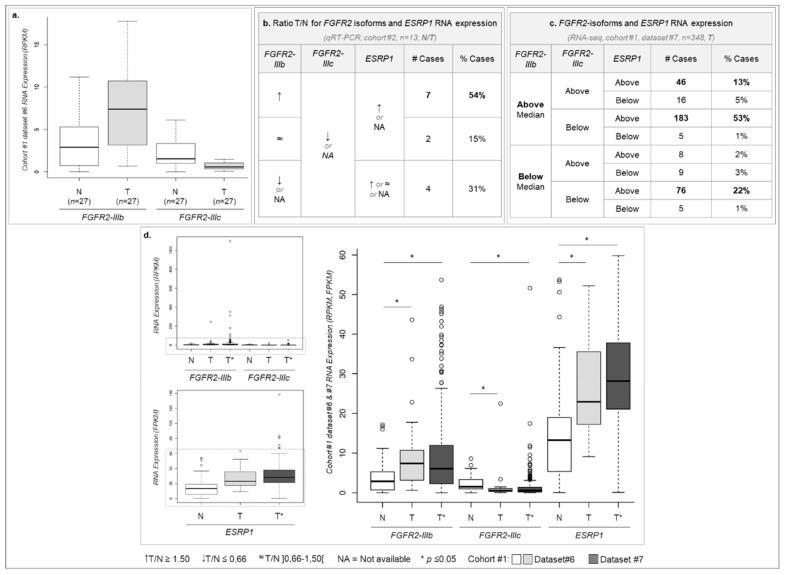
*FGFR2* isoforms and *ESRP1* RNA expression status across several cohorts. (**a**) Cohort #1 dataset #6 RNA expression for 27 paired normal (white) and tumor (grey) samples for the specific exons *FGFR2-IIIb* and *FGFR2-IIIc*. (**b**) Table with the number and percentage of cases with a given RNA expression profile for *FGFR2-IIIb*, *FGFR2-IIIc* and *ESRP1* in 13 GC paired cases from cohort #2, determined by qRT-PCR. Upwards arrow for cases where the expression ratio (T/N) is equal or above 1.50, downwards arrow for cases with expression ratio below 0.67, and ‘~’ when the expression ratio is between 0.67 and 1.5. NA stands for not available. (**c**) Table with the number of cases with a given RNA expression for *FGFR2-IIIb*, *FGFR2-IIIc* and *ESRP1* in 348 GC samples (unpaired) from cohort #1 dataset #7, determined by *RNA-seq*. Due to the absence of paired normal samples in cohort #1 dataset #7, the median RPKM value for each transcript in cohort #1 dataset #6 normal samples was used as threshold to determine the number of cases with RNA expression above or below it in cohort #1 dataset #7. Median RNA expression values calculated for normal stomach samples were: *FGFR2-IIIb* = 2.89; *FGFR2-IIIc* = 1.53; *ESRP1* = 13.26. (**d**) Boxplot representation of the RNA expression of the specific exons for *FGFR2-IIIb* and *FGFR2-IIIc* isoforms (RPKM) and for the canonical *ESRP1* transcript (FPKM) for the paired normal and tumor cases from cohort #1 dataset #6 (N and T) and the unpaired tumor cases from cohort #1 dataset #7 (T*). The larger boxplot is a zoom in from the region represented with dotted rectangles in the smaller boxplots. Asterisks stand for *p*-value ≤ 0.05. Of notice, *ESRP1* RNA expression data are the same as represented in Supplementary Figure S4j.

Given the role of ESRP1 as the main regulator of *FGFR2* alternative splicing, we next calculated the expression of *FGFR2-IIIb* and *FGFR2-IIIc* specific exons, as surrogates of the respective *FGFR2* isoforms (Figure 2a and Material and Methods Section). We then correlated *FGFR2* isoform expression with the previously described *ESRP1* expression (Figure S4c), in T*vs*N and unpaired tumor datasets from TCGA (cohort #1 datasets #6 and #7). *FGFR2-IIIb* expression was higher in tumors than in normal tissue, while the opposite occurred for *FGFR2-IIIc* (Figure 2a, *p*-value = 9.46 × 10^−3^ and 1.90 × 10^−3^, respectively). This result was mimicked in TvsN cases from cohort #2 (Figure 2b). Given that there were only 27 TvsN pairs for expression analysis in TCGA, we calculated the median expression detected in the 27 normal samples (cohort #1 dataset #6) and used it for comparison with data from 348 tumor samples (cohort #1 dataset #7). More than half (183/348—53%) of tumor samples presented *FGFR2-IIIb* and *ESRP1* overexpression and *FGFR2-IIIc* under-expression in comparison to the median expression of normal stomach samples (Figure 2c). By comparing exact RNA expression values, instead of using the median, we observed that the expression of *FGFR2* isoforms and *ESRP1* is consistent in tumors from two different datasets (cohort #1 datasets #6 and #7) (*p*-value > 0.05, Figure 2d), while being significantly distinct from the expression of both genes detected in normal samples (cohort #1 dataset #6, *p*-value ranging from 8.93 × 10^−6^ to 9.46 × 10^−3^, Figure 2d). Overall, in comparison with normal stomach, GC tumors express high RNA levels of *FGFR2-IIIb* and *ESRP1* and low *FGFR2-IIIc* RNA levels.

### 2.4. Expression of ESRP1 and FGFR2 Isoforms Are Significantly Correlated with CN Status of Corresponding Gene Loci in GC

**Figure 3:**
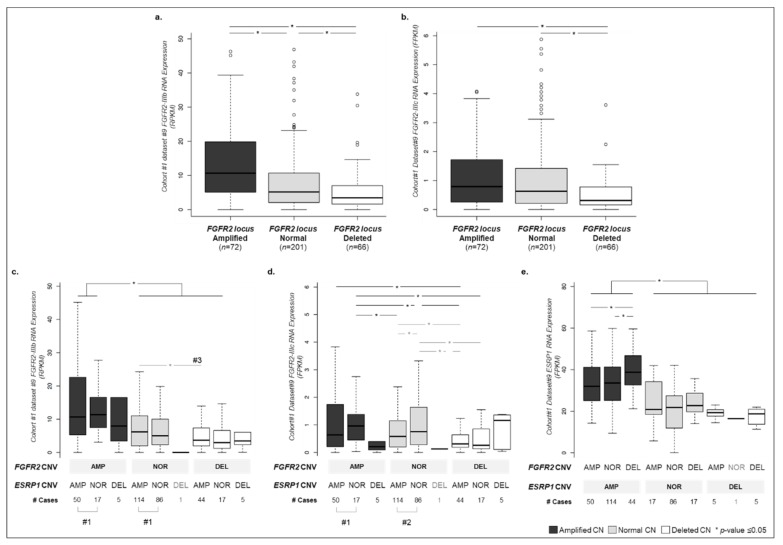
Copy number status of *FGFR2* isoforms and *ESRP1* and corresponding RNA expression level across GC cohort #1 dataset #9. (**a**) *FGFR2-IIIb*-specific exon RNA expression (RPKM) for GC cohort #1 dataset #9 tumor samples (*n* = 339) separated according to *FGFR2* somatic copy number status: amplified (dark grey), normal (grey) and deleted (white). Not all outliers are displayed. (**b**) Same as (**a**) for the RNA expression of *FGFR2-IIIc* specific exon (RPKM). (**c**) Same samples represented in (**a**) and (**b**) re-organized according to the copy number status of both *FGFR2* and *ESRP1 loci*. All 9 possible combinations for amplified (AMP), normal (NOR) and deleted (DEL) were detected in GC cohort #1 dataset #9 samples and are described in the X-axis along with the number of cases observed. Not all outliers are displayed. RNA expression of *FGFR2-IIIb* specific exon is depicted. (**d**) Same as (c) for *FGFR2-IIIc* specific exon. (**e**) Same as (**c**) for *ESRP1* transcript and as in Figure S4j.

To verify if there was an association between the expression of *FGFR2* isoforms and *FGFR2* and *ESRP1* CN status, we analyzed cohort #1 dataset #9. *FGFR2-IIIb* expression was significantly increased in *FGFR2* amplified cases, in comparison with tumors where *FGFR2* was normal or deleted (*p*-value = 4.05 × 10^−5^, Figure 3a). Moreover, *FGFR2-IIIb* expression was directly correlated with *FGFR2* CN status, i.e., amplified cases presenting the highest RNA expression, while deleted cases presenting the lowest RNA expression (*p*-value ranging from 3.10 × 10^−7^ to 1.52 × 10^−2^, Figure 3a). This was also generally true for *FGFR2-IIIc* (Figure 3b).

To understand whether *ESRP1* CN status was correlated with *FGFR2* isoform expression, we categorized each case according to both *FGFR2* and *ESRP1* CN status and analyzed the expression of *FGFR2* isoforms. In tumors with *ESRP1* amplification or normal CN, *FGFR2-IIIb* expression was similar, even if *FGFR2* was amplified (Figure 3c, #1, Figure S5). This was also valid for *FGFR2-IIIc* expression exclusively in *FGFR2* amplified cases (Figure 3d, #1, Figure S5). In tumors with normal *ESRP1* and *FGFR2* CN, *FGFR2-IIIc* expression was higher than in tumors with *ESRP1* amplification (Figure 3d, #2, Figure S5). Moreover, only if *ESRP1* was amplified, *FGFR2-IIIb* expression was significantly higher in cases with normal *FGFR2* CN in comparison with cases with *FGFR2* deletion (Figure 3c, #3, Figure S5). We also verified that high *ESRP1* expression is mainly driven by its own amplification and does not depend on *FGFR2* CN status (Figure 3e).

Overall, these data support that in GC, ESRP1 CN changes are major regulators of not only its own expression but also of FGFR2 isoforms, favoring FGFR2-IIIb in opposition to FGFR2-IIIc expression.

### 2.5. Patients with Low FGFR2-IIIc Expression and Diffuse-Type GC Present Better Overall Survival than Those with FGFR2-IIIc High Expression

Given the expressional differences between normal and tumor samples for *FGFR2* isoforms and *ESRP1*, we next crossed these data with several clinico-pathological features made available by the TCGA consortium. In particular, we have categorized every GC sample (cohort #1 dataset #11) as displaying *FGFR2-IIIb*, *FGFR2-IIIc* and *ESRP1* expression above or below the median expression detected in normal samples (cohort #1 dataset #6). As control, the same categorization was performed taking into account the RNA expression of the shared up/downstream exons of *FGFR2*. 

We found that GC patients whose tumors present low *FGFR2-IIIc* expression (below the median) were more frequently alive (*p*-value = 1.46 × 10^−2^), and tumors were often of the intestinal type (*p*-value = 1.21 × 10^−6^), preferentially from the CIN (chromosomal instable) subgroup and rarely genomically stable (GS) (*p*-value = 3.54 × 10^−7^) (Table 1) [18]. In contrast, GC patients whose tumors presented high *FGFR2-IIIc* expression (above the median) were more frequently of the diffuse type and belonged to the GS subgroup. Concerning *ESRP1* expression, 85% of tumors presenting low *ESRP1* expression (below the median) were of the diffuse type (*p*-value = 3.14 × 10^−10^) and 80% belonged to the GS subgroup (*p*-value = 1.07 × 10^−7^), while those presenting high *ESRP1* expression, were mainly of the intestinal type and CIN subgroup. No statistically significant associations were identified between *FGFR2-IIIb* expression and clinico-pathological features of patients and tumors.

**Figure 4:**
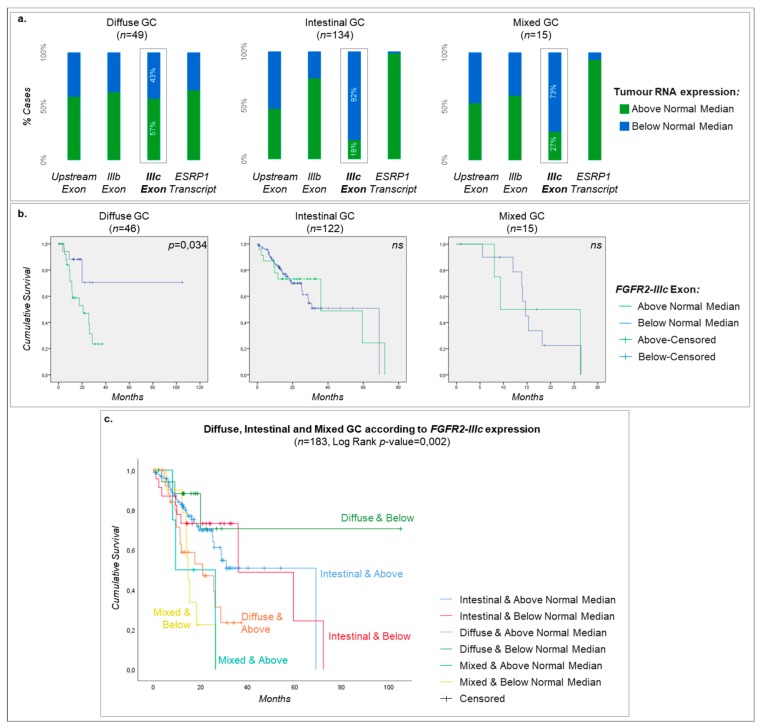
Correlation between *FGFR2-IIIc* expression and clinico-pathological features. (**a**) Percentage of GC (cohort #1 dataset #11) divided according to the Lauren Classification (Diffuse, Intestinal or Mixed) displaying RNA expression of *FGFR2* upstream and downstream exons, *FGFR2-IIIb* or *FGFR2-IIIc* specific exons above (green) or below (blue) the normal stomach median expression. (**b**) Individual Kaplan-Meier plots for diffuse, intestinal or mixed GC separated according to *FGFR2-IIIc* specific exon expression (above/below the median of normal stomach). (**c**) Kaplan-Meier plot for diffuse, intestinal or mixed GC separated according to *FGFR2-IIIc* specific exon expression (above/below the median of normal stomach).

With this analysis, we also observed for cohort #1 dataset #11 that different histological types presented distinct above/below distribution, particularly for *FGFR2-IIIc*. While most intestinal and mixed-type GCs presented low *FGFR2-IIIc* expression, for the diffuse-type cases a 50–50 proportion was observed (Figure 4a). Given the well-known correlation between diffuse-type GC and worse prognosis, we next performed a survival analysis. Indeed, this analysis showed that patients with diffuse-type GC presenting high *FGFR2-IIIc* expression had a significantly worse overall survival (Figure 4b,c, overall log-rank *p*-value = 3.40 × 10^−2^, for all comparisons see Figure S6a). The same was not observed for intestinal and mixed-type GCs, neither for *FGFR2-IIIb*, *ESRP1* nor *FGFR2-IIIc* (data not shown). The same biased distribution for *FGFR2-IIIc* expression above/below normal stomach median was observed exclusively in GS GCs (Figure S7b). This also translated into a worse overall survival of GC patients with GS and high *FGFR2-IIIc* expression (Figure S7c, *p*-value = 2.4 × 10^−2^, log-rank test). Importantly, none of these associations with overall survival could be attributed to tumor stage, given that this cohort presented similar frequencies of stage I/II and stage III/IV tumors in each category of RNA expression (Table 1).

To understand if *FGFR2*/*ESRP1* CN status could also be correlated with overall survival, the same clinico-pathological factors were studied (Table S3). We observed that there was a significant correlation between tumor histotype and *ESRP1* CN (*p*-value = 2.55 × 10^−3^): while most amplified tumors were of the intestinal type, almost 40% of samples with normal *ESRP1* CN were diffuse-type GCs. We also saw that the majority of tumors with *FGFR2* or *ESRP1* amplification or deletion were of the CIN subtype (*p*-value = 2.82 × 10^−12^ and 2.58 × 10^−4^, respectively).

We next performed a survival analysis; however no significant differences were found for tumors with different *FGFR2* or *ESRP1* CN status, even when taking into account the histotype or the tumor stage (Figure S7).

### 2.6. ESRP1 Control over FGFR2 Isoform Expression May Be GC Histotype-Dependent

**Figure 5:**
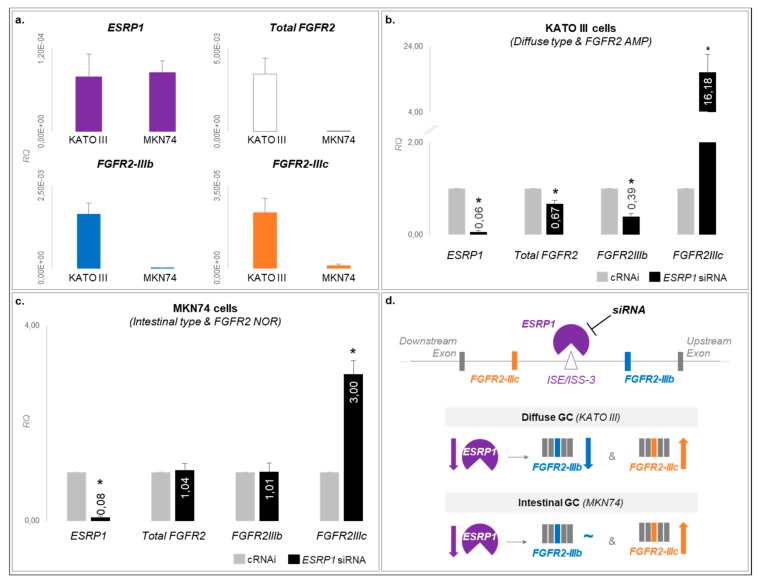
*ESRP1* controls *FGFR2* isoform expression in a distinct manner according to GC cell line histotype. (**a**) *ESRP1*, total *FGFR2*, *FGFR2-IIIb* and *FGFR2-IIIc* RNA expression in parental KATO-III (diffuse type) and MKN74 (intestinal type) GC cell lines. RQ stands for relative quantification. (**b**) *ESRP1*, total *FGFR2*, *FGFR2-IIIb* and *FGFR2-IIIc* RNA expression in control cells (grey) and *ESRP1*-siRNA-treated KATO-III cells (black). Asterisk stand for *p* < 0.05. (**c**) Same as (**b**) for control cells (grey) and *ESRP1*-siRNA-treated MKN74 cells. (**d**) Summary model of the differences observed between the diffuse- and intestinal-type GC cell lines in terms of *FGFR2* isoform expression upon *ESRP1* expression inhibition.

Given the specific association between high expression of *FGFR2-IIIc* and poorer overall survival, specifically for diffuse GCs, and the known role of ESRP1 in controlling *FGFR2* splicing, we hypothesized that this control could occur differently depending on the GC histotype. To test this, we used a diffuse and an intestinal gastric cancer cell line: KATO-III and MKN74, respectively. Of notice, KATO-III parental cells already presented very high expression levels of total *FGFR2* and both isoforms, due to a known *FGFR2* amplification, unlike MKN74 parental cells (Figure 5a). Using RNAi, we depleted *ESRP1* expression (>90% efficiency, Figure 5b–d), and observed that total *FGFR2* and *FGFR2-IIIb* RNA expression significantly decreased specifically in KATO-III cells (Figure 5b), while *FGFR2-IIIc* RNA expression significantly increased in both KATO-III and MKN74 cells (Figure 5c). These differences supported our hypothesis that ESRP1 plays a different role in distinct GC histological types regarding splicing/expression regulation of *FGFR2* isoforms, and that the effect is more pronounced in the diffuse-type GC.

## 3. Discussion

The objective of this study was to explore the mechanisms dysregulating the expression of *FGFR2* and its splicing regulator *ESRP1* in GC, by analyzing changes in copy number, promoter methylation and RNA expression of *FGFR2* and its isoforms. This knowledge is expected to shed light into novel predictive biomarkers for stratification of GC patients for anti-FGFR2 therapy.

We first explored CNVs in both *FGFR2* and *ESRP1 loci* and found that these genes were frequently amplified or co-amplified in gastric tumors. Albeit increased CN of these *loci* has been previously reported [17,18,46], the same is not true for their co-amplification. We also observed that tumors with *FGFR2* and *ESRP1* genetic amplification presented increased RNA expression of the respective gene, supporting CN change as one of the mechanisms underlying *FGFR2*/*ESRP1* signaling dysregulation in GC.

We next explored whether the *FGFR2* promoter methylation status could further explain its overexpression in GC: we observed that most tumors displaying low levels of *FGFR2* promoter methylation showed increased RNA expression than those with higher methylation levels. This was also true for *ESRP1*, which displayed low levels of promoter methylation in almost all tumor samples and higher RNA expression than those with other methylation levels. These data are consistent with the expected control over RNA expression exerted by methylation at CpG islands [50,51] and indicate that both *FGFR2* and *ESRP1* promoters, by being overall demethylated, are likely in a transcriptionally permissive state.

We also found that the expression of *FGFR2-IIIb*, but not of total *FGFR2*, was significantly increased in tumor samples. This was expected given the known role of ESRP1 in the regulation of *FGFR2* alternative splicing. Our data is in accordance with previously published studies reporting the prevalence of the *FGFR2-IIIb* isoform in GC [40,41], although further validation at the protein level should be performed. Interestingly, we found that the increase in *FGFR2-IIIb* expression occurred concomitantly with a decrease in *FGFR2-IIIc* expression in GC. This *bias* towards *FGFR2-IIIb* is particular to stomach tumors, as both isoforms present similar RNA expression levels in normal stomach, revealing a tight control of this process in normal tissue as opposed to cancer. This result also suggests that the expression of *FGFR2* isoforms is controlled in a tissue- and cancer-type specific manner [39]. For example, in normal kidney *FGFR2-IIIb* is overexpressed in detriment of *FGFR2-IIIc*, while in clear cell renal cell carcinoma (ccRCC), *FGFR2-IIIc* becomes overexpressed in detriment of *FGFR2-IIIb* [39].

We next tried to understand whether the pattern of expression of *FGFR2* isoforms in GC was correlated with *FGFR2* and *ESRP1* CN status and discovered that *FGFR2-IIIb* was significantly increased in tumors with *FGFR2* amplification (Figure 3a,b). Interestingly, this genetic alteration was not associated with low *FGFR2-IIIc*, which could indicate that only *FGFR2-IIIb* is selectively dysregulated in *FGFR2*-amplified GC tumors. However, when considering the CN status of both genes, we observed that the *ESRP1* CN affected only *FGFR2-IIIc* RNA expression (Figure 3d). We observed that in tumors with *FGFR2* normal CN, *ESRP1* amplification was associated with a significant decrease in *FGFR2-IIIc* RNA expression in comparison with tumors with normal *ESRP1* CN (Figure 3e, #2). Although it has been previously reported that *FGFR2-IIIc* down-regulation is due to *ESRP1* overexpression [52], our study is the first to reveal the genetic mechanism by which *ESRP1* becomes overexpressed (gene amplification) promoting *FGFR2* isoform expression *bias*. This data represents a novel layer in the expression regulation of *FGFR2* isoforms, and supports further studying *FGFR2-IIIc* dysregulation in tumors with *FGFR2* normal CN.

The relevance of *FGFR2-IIIc* expression in GC was further emphasized by important correlations found with clinico-pathological data and the overall survival of patients. Indeed, we verified that GCs of the diffuse histological type presenting high *FGFR2-IIIc* RNA expression presented significantly poorer overall survival than those with low expression. Not surprisingly, the same was observed in GS GCs, as this molecular subtype is known to greatly overlap diffuse-type GC [50]. Strikingly, for *FGFR2-IIIb* no particular correlations were identified, contrarily to previous reports [40,53]. For example, Ahn et al. (2016) showed that patients with diffuse-type GC and FGFR2-IIIb protein overexpression presented better overall survival [40]. Although this could be related to our lack of protein data, it may also be due to the lack of normal samples analyzed by Ahn et al. In fact, non-cancerous gastric tissue has been shown to display FGFR2-IIIb staining [54], providing relevance to our choice of using the median expression levels detected in normal samples as a cut-off for gene/isoform overexpression,. Han et al. also showed a strong association between *FGFR2-IIIb* RNA and protein expression, strengthening the confidence in our approach. We also confirmed that our latter results were not biased by an uneven distribution of stage III/IV tumors among *FGFR2-IIIc*-overexpressing tumors, which despite needing independent validation, strengthens the value of our findings.

Supporting previous reports showing a lack of correlation between *FGFR2* CN and the overall survival of patients [12], we also found no correlation for *FGFR2* and *ESRP1* CN status in gastric tumors and overall survival of patients. 

As a final experiment to understand the regulation of ESRP1 over *FGFR2* isoforms in an histotype-dependent manner, we depleted *ESRP1* in GC-derived cell lines. ESRP1 was only capable of regulating both *FGFR2* isoforms in a diffuse GC cell line (Figure 5d). As diffuse-type GC is known to be associated with a more stem-cell-like signature [55], our in vitro results with KATO-III cells recall those by Fagoonee et al., showing that ESRP1-knockdown in mouse embryonic stem-cells also led to an expression *bias* towards *FGFR2-IIIc* [56].

Overall, our results encourage further studies on the role of *ESRP1* in GC and support *FGFR2-IIIc* as a relevant biomarker in this disease.

## 4. Materials and Methods 

### 4.1. Description of Cohorts

In this study, three main data categories were used: copy number variation (CNV), DNA methylation, and transcriptome profiling. A total of 4 GC cohorts were used for our study: cohort #1 from TCGA; cohort #2, a private GC cohort; cohort #3 from Kwon et al. [57] and; cohort #4 from Lei et al. [55] (Table S1). In particular, cohort #1 was split in 11 datasets depending on the data category assessed: dataset #1–338 tumors analyzed for CNV by Affymetrix SNP 6.0 array; dataset #2–91 normal/tumor pairs analyzed for CNV by Affymetrix SNP 6.0 array; dataset #3–27 normal/tumor pairs analyzed for DNA methylation with *Illumina Human Methylation 27* beadchip; dataset #4–416 tumors analyzed for DNA methylation with *Illumina Human Methylation 450k* beadchip; dataset #5–376 tumors analyzed for CNV by Affymetrix SNP 6.0 array and DNA methylation with *Illumina Human Methylation 450k* beadchip; dataset #6–27 normal/tumor pairs analyzed for transcriptome profiling by RNA-sequencing; dataset #7–348 tumors analyzed for transcriptome profiling by RNA-sequencing; dataset #8–23 normal/tumor pairs analyzed for CNV by Affymetrix SNP 6.0 array and for transcriptome profiling by RNA-sequencing; dataset #9–339 tumors analyzed for CNV by Affymetrix SNP 6.0 array and for transcriptome profiling by RNA-sequencing; dataset #10–375 tumors analyzed for DNA methylation by *Illumina Human Methylation 27* or *450k* beadchip and for transcriptome profiling by RNA-sequencing and; dataset #11–198 tumors analyzed for CNV and transcriptome profiling with relevant clinical data, particularly concerning patient (gender, age, race, ethnicity, age at diagnosis, vital status, days to death if applicable) and the tumor (stage, Lauren class, and molecular subtype). Cohort #2 entailed 47 paired normal mucosa and gastric tumors analyzed for CNV by Whole-Genome Sequencing (WGS, Complete Genomics platform performed as a service by BGI, Shenzhen, China) and DNA methylation by Reduced Representation Bisulfite Sequencing (*RRBS*, Illumina platform, performed as a service by BGI, Shenzhen, China). Cohort #3 encompassed 32 normal/tumor pairs analyzed for DNA methylation with *Illumina Human Methylation 27* beadchip (GSE25869, [57]). Cohort #4 was constituted by 75 normal/tumor pairs analyzed for DNA methylation with *Illumina Human Methylation 27* beadchip (GSE30601 [55]). In Table S2 it is possible to observe the overlap between cohort #1 samples across the distinct datasets #1-10.

### 4.2. Copy Number Variation Data Analysis

For cohort #1 datasets #1, #2, #5, #8, #9 and #11, CNV (masked) was obtained in terms of segment mean values, downloaded from the Genomic Data Commons (GDC) data portal [58], particularly for samples in the TCGA-STAD project. As performed by Laddha et al. [59], we defined the segment mean cut-offs by analyzing *FGFR2* and *ESRP1* distribution of segment mean values, for all available normal mucosa and gastric tumor samples (datasets #1, #2, Figure S1). This analysis showed that a cut-off of ±0.1 segment mean was enough to separate normal from tumor samples for both genes. Therefore, we classified *FGFR2*/*ESRP1* as: (1) amplified when the segment mean was above 0.1; (2) deleted when the segment mean was below -0.1 and; (3) with normal copy number when the segment mean was between −0.1 and 0.1. Furthermore, only samples for which all probes overlapping *FGFR2* or *ESRP1 loci* were concordant were considered. For each of cohort #2 paired samples, DNA was extracted using QIAamp DNA Mini Kit (Qiagen) and subjected to WGS as a service by BGI (Shenzhen, China) using the Complete Genomics platform. Resulting data was analyzed using BGI internal pipelines and GISTIC 2.0 [60] to determine CNV in normal and tumor pairs from cohort#1. Genes with GISTIC 2.0 values equal or above 1 were considered to be amplified, genes with values equal or below -1 as deleted and genes with values equal to 0 as without CNV.

### 4.3. DNA Methylation Data Analysis

#### 4.3.1. RRBS

For each of cohort #2 paired samples, extracted DNA was subjected to RRBS as a service by BGI (Shenzhen, China) using an Illumina platform. Resulting data was analyzed using BGI internal pipelines [61] and the methylation levels for *FGFR2* and *ESRP1* promoters across cohort #1 normal and tumor paired samples was retrieved. The promoters of *FGFR2* and *ESRP1* were defined by BGI internal pipelines as the region ranging from the TSS to 2000 bp upstream of it: *FGFR2* promoter was localized at chr10:121598458-121600598 and *ESRP1* promoter at chr8:94639136-94641136 (UCSC genome browser, hg38 [62]). Next, we calculated the ratio of the methylation level for each normal/tumor pair from cohort #1, and: if the ratio was equal or higher than 1.5, the sample was classified as hypermethylated; if the ratio was below or equal to 0.66, the sample was classified as hypomethylated (i.e., 2-fold decrease in the tumor counterpart); if the ratio was between 0.66 and 1.5, the sample was classified as normal.

#### 4.3.2. Bisulfite Sanger Sequencing

DNA from 13 normal/tumor pairs from cohort #2 was bisulfite-converted using the Epitect Bisulfite Kit (Qiagen) following manufacturer’s instructions. Afterwards, *FGFR2* and *ESRP1* promoter methylation status was validated using two pairs of primers designed to amplify bisulfite-treated DNA in the regions defined by the coordinates chr10:121598809-121598954 and chr8:94640249-94640436 (*FGFR2* and *ESRP1* respectively, UCSC genome browser, hg38 30), selected as proxies for the promoter regions evaluated by RRBS. For *FGFR2* promoter proxy, the primers used were: 5′-GGGAGGGTAGGGTTAGAG-3′ and 5′-CCCTCTCTACCAATCAAC-3′. Up to 36 CpG sites could be detected however consistent results were only observed for CpG sites 5-24. For *ESRP1* promoter proxy, the primers used were 5′-GGAGTGATTAGGTGGTTGG-3′ and 5′-CAACTCCTAAACCAACACAAC-3′.

#### 4.3.3. Illumina Human Methylation 27/450 Beadchip Data Collection

For cohort #1 datasets #3, #4, #5, #10, the beta-values for normal/tumor samples obtained using these chips was collected from the TCGA project, using the GDC data portal and samples from the TCGA-STAD project. For cohorts #3 and #4, the beta-values for the GEO-deposited datasets GSE25869 and GSE30601. For cohort #2 dataset #3 and cohorts #3 and #4 intensity data was available for both paired normal and tumor samples only for 2 probes overlapping *FGFR2* and 1 probe overlapping *ESRP1* CpG islands and selected proxies. For *FGFR2*, intensity values were collected for probes cg17028039 and cg09772154. For *ESRP1*, intensity values were collected for probe cg26350286. For cohorts 7 and 12, intensity information was available for up to 9 probes overlapping *FGFR2* and 3 probes overlapping *ESRP1* CpG islands and selected proxies. For *FGFR2*, intensity values were collected for probes cg03471571, cg05368033, cg12835048, cg17028039, cg06657142, cg17794169, cg22762615, cg02179499, and cg09772154. For *ESRP1*, intensity values were collected for probes cg14154651, cg26350286, and cg07473471. The average beta-value for available probes was calculated and given that the average beta-value values range from 0 (fully demethylated) to 1 (fully methylated), samples were classified as: demethylated, if the average beta-value was equal or below 0.33; hemimethylated, if the average beta-value was between 0.33 and 0.66 and; methylated, if the average beta-value was equal or above 0.66.

### 4.4. Transcriptome Profiling Analysis

#### 4.4.1. FGFR2, ESRP1, FGFR2-IIIb, and FGFR2-IIIc Relative Quantification

RNA was extracted from 13 normal/tumor pairs from cohort#1 using the mirVANA Isolation Kit (Thermo Fisher Scientific, Waltham, MA, USA) according to manufacturer instructions for total RNA isolation. cDNA was generated using SuperScript II Reverse Transcriptase (Thermo Fisher Scientific) and all related reagents, following manufacturer instructions. Next, quantitative real-time PCR was performed using Kapa Probe Fast qPCR Master Mix (Roche, Basel, Switzerland) and related reagents following manufacturer instructions. The pre-designed PrimeTime qPCR assays Hs.PT.58.1565679 and Hs.PT.58.24361486 (IDT) were used for total *FGFR2* and *ESRP1* quantification with the 18S TaqMan probe Hs99999901_s1(Thermo Fisher Scientific) as housekeeping gene. For *FGFR2-IIIb* the custom designed assays included: probe 5′-AACAGCAAG/ZEN/CGCCTGGAAGAGAAA-3′; primer 1 5′-CAATTATATAGGGCAGGCCAAC-3′; primer 2 5′-CCCTATGCAGTAAATGGCTATC-3′. For *FGFR2-IIIc* the custom designed assays included: probe 5′-TCTGCATGG/ZEN/TTGACAGTTCTGCCA -3′; primer 1 5′-CTTGGCGGGTAATTCTATTGG-3′; primer 2 5′-CCCTATGCAGTAAATGGCTATC-3′.

#### 4.4.2. RNA-Sequencing Data Analysis

Using the original data files for cohort#1 datasets #5 to #10, we specifically retrieved the FPKM values for the *FGFR2* and *ESRP1* genes, a value which combines the expression information for all annotated transcripts (FPKM). In addition, we retrieved the RPKM values for the following specific FGFR2 exons (Figure S2): (1) *FGFR2-IIIb* specific exon, which corresponds to exon number 8 for transcript NM_022970 or ENST00000457416; (2) *FGFR2-IIIc* specific exon, which corresponds to exon number 6 for transcript NM_001144916 or ENST00000356226; (3) the closest upstream exon for both transcripts, i.e., exon number 7 or exon number 5 for *FGFR2-IIIb* or *FGFR2-IIIc* transcripts, respectively; (4) the closest downstream exon, i.e., exon number 9 or exon number 7 for *FGFR2-IIIb* or *FGFR2-IIIc* transcripts, respectively). 

### 4.5. Cell Culture and Short-Interference-RNA Experiments

Gastric cancer cell lines MKN74 and KATO-III cell lines (from ATCC) were cultured using recommended mediums: RPMI 1640 culture medium (Gibco, Gaithersburg, MD, USA) supplemented with 10% fetal bovine serum (Biowest, Nuaillé, France) and 1% penicillin-streptomycin (Invitrogen, Carlsbad, CA, USA). Near-normal mammary epithelial cells MCF10A were cultured in DMEM/F12 Glutamax medium (Gibco) supplemented with 5% horse serum (Lonza, Basel, Switzerland), 5 mg/mL recombinant human insulin (Sigma-Aldrich, St. Louis, MO, USA), 1% penicillin-streptomycin (Invitrogen), 500 ng/mL hydrocortisone (Sigma-Aldrich), 20 ng/mL cholera toxin (Sigma-Aldrich) and 20 ng/mL recombinant human epidermal growth factor (Sigma-Aldrich). All cell lines were kept in culture flasks at approximately 37 °C and 5% CO_2_. All cell lines authentication was performed at the Ipatimup’s Cell Lines Bank, using STR amplification (Promega-Powerplex16, Identifiler, Carnaxide, Portugal). Cells were treated with human short-interference-RNA ESRP1 siGENOME-SMARTpool at 50 nM for 72 h (Thermo Fisher Scientific) or ON-TARGET plus non-targeting siRNA #4 at 50 nM for 72 h (Thermo Fisher Scientific) as non-targeting control. Lipofectamine 2000 (Thermo Fisher Scientific) was used as transfection agent. Afterwards RNA was extracted followed by *FGFR2* (total and isoforms) and *ESRP1* RNA quantification as described previously.

### 4.6. Graphical Representations and Statistical Analysis

All density plots and boxplots presented were performed using R and the package “ggplot2” [63,64]. Statistical analyses were performed also using R, in particular the nonparametric Wilcoxon rank-sum test. Student’s t-test was used for the analysis presented in Figure 5b,c.

## 5. Conclusions

Our study provides the first in-depth analysis of copy number and promoter methylation as the mechanisms dysregulating the expression of total *FGFR2*, its splicing regulator *ESRP1* and the *FGFR2-IIIb* and *FGFR2-IIIc* isoforms in GC.

We unveiled for the first time a link between *ESRP1* amplification and *FGFR2-IIIc* high expression, through the axis ESRP1^amp^-FGFR2^norm^-FGFR2-IIIc^high^, which seems to particularly determine the poor overall survival of patients with diffuse-type GC. These results raise the importance of evaluating, particularly in diffuse-type GC, the expression of *FGFR2-IIIc*, rather than *FGFR2-IIIb* or total *FGFR2*. Therefore, we believe *FGFR2-IIIc* should be explored as a molecular target for patients with diffuse-type GC, also providing an opportunity to repurpose available anti-FGFR2-IIIc therapies. Moreover, *FGFR2-IIIc* RNA expression may constitute a useful predictive marker of therapy response, not only for anti-FGFR2-IIIc therapies but also for other anti-FGFR2 or anti-FGFR2-IIIb therapies currently in clinical trials (e.g., #NCT03694522 based on FGFR2-IIIb overexpression).

## Figures and Tables

**Table 1:** Clinico-pathological factors and expression of *FGFR2* distinct exons and *ESRP1* in cohort #1 dataset #11 gastric tumors.

Clinico-Pathological Factor		*FGFR2* RNA Expression ^1^						*ESRP1* RNA Expression ^1^	
		Upstream Exon		Specific Exon IIIB		Specific Exon IIIC			
		Above (n = 100)	Below (n = 98)	Above (n = 141)	Below (n = 57)	Above (n = 56)	Below (n = 142)	Above (n = 178)	Below (n = 20)
**Gender**	Female	36 (36%)	38 (39%)	53 (38%)	21 (37%)	21 (38%)	53 (37%)	66 (37%)	8 (40%)
	Male	64 (64%)	60 (61%)	88 (62%)	36 (63%)	35 (63%)	89 (63%)	112 (63%)	12 (60%)
	*p*-value	ns		ns		ns		ns	
**Age**	< 65	44 (44%)	42 (43%)	62 (44%)	24 (42%)	30 (54%)	56 (39%)	71 (40%)	12 (60%)
	>=65	56 (56%)	56 (57%)	79 (56%)	33 (58%)	26 (46%)	86 (63%)	103 (58%)	8 (40%)
	*p*-value	ns		ns		ns		ns	
**Vital Status**	Dead	41 (41%)	30 (31%)	51 (36%)	20 (35%)	28 (50%)	43 (30%)	62 (35%)	9 (45%)
	Alive	59 (59%)	68 (69%)	90 (64%)	37 (65%)	28 (50%)	99 (70%)	116 (65%)	11 (55%)
	*p*-value	ns		ns		1.46 × 10^−2^		ns	
**Lauren Class.**	Diffuse	29 (29%)	20 (20%)	31 (22%)	18 (32%)	28 (50%)	21 (15%)	32 (18%)	17 (85%)
	Intestinal	63 (63%)	71 (72%)	101 (72%)	33 (58%)	24 (43%)	110 (77%)	132 (74%)	2 (10%)
	Mixed	8 (8%)	7 (7%)	9 (6%)	6 (11%)	4 (7%)	11 (8%)	14 (8%)	1 (5%)
	*p*-value	ns		ns		1.21 × 10^−6^		3.14 × 10^−10^	
**Stage**	I/II	46 (46%)	49 (50%)	67 (48%)	28 (49%)	29 (52%)	66 (46%)	88 (49%)	7 (35%)
	III/IV	46 (46%)	41 (42%)	60 (43%)	27 (47%)	24 (43%)	63 (44%)	76 (43%)	11 (55%)
	NA	8 (8%)	8 (8%)	14 (10%)	2 (4%)	3 (5%)	13 (9%)	14 (8%)	2 (10%)
	*p*-value	*ns*		*ns*		*ns*		*ns*	
**Molecular Subtype**	CIN	51 (51%)	50 (51%)	74 (52%)	27 (47%)	19 (34%)	82 (58%)	97 (54%)	4 (20%)
	EBV	7 (7%)	10 (10%)	12 (9%)	5 (9%)	2 (4%)	15 (11%)	17 (10%)	0 (0%)
	MSI	18 (18%)	19 (19%)	26 (18%)	11 (19%)	8 (14%)	29 (20%)	37 (21%)	0 (0%)
	GS	24 (24%)	19 (19%)	29 (21%)	14 (25%)	27 (48%)	16 (11%)	37 (21%)	16 (80%)
	*p*-value	ns		ns		3.54 × 10^−7^		1.07 × 10^−7^	

^1^ Percentages calculated in relation to total number of cases displayed on the ‘Above’ or ‘Below’ categories.

## Supplementary Materials

The following are available online at https://www.mdpi.com/2072-6694/12/1/70/s1, Table S1: Description of the data category, sample type, number of samples and source for each of the gastric cancer cohorts/datasets described in this study. Table S2: Description of the data category, sample type, number of samples, source and sample overlap for each of the 11 gastric cancer datasets derived from TCGA described in this study (cohort #1). Table S3: Clinico-pathological factors and distinct CNVs detected in FGFR2 and ESRP1 loci across cohort #1 dataset #11 gastric tumors. Figure S1: Distribution of gene segment mean values in cohort #1 dataset #1 and dataset #2 samples. Figure S2: Representation of the alternative splicing underlying the two FGFR2 isoforms studied: FGFR2-IIIb and FGFR2-IIIc. Figure S3: Schematic representation of FGFR2 and ESRP1 promoter regions evaluated by Bisulfite Sanger sequencing. Figure S4: Total FGFR2 and ESRP1 RNA expression, CNV and promoter methylation status. Figure S5: Significance matrices for RNA expression of *FGFR2-IIIb*, *FGFR2-IIIc* and *ESRP1* in cohort #1 dataset #9 GC cases separated and compared according to *FGFR2* and *ESRP1* CN status. Figure S6: Correlation between *FGFR2-IIIc* expression and clinico-pathological features. Figure S7: Correlation between *FGFR2* and *ESRP1* CN and clinico-pathological features.

## References

[1] Bray F., Ferlay J., Soerjomataram I., Siegel R.L., Torre L.A., Jemal A. (2018). Global cancer statistics 2018: GLOBOCAN estimates of incidence and mortality worldwide for 36 cancers in 185 countries. CA Cancer J. Clin..

[2] Balakrishnan M., George R., Sharma A., Graham D.Y. (2017). Changing Trends in Stomach Cancer Throughout the World. Curr. Gastroenterol. Rep..

[3] Karimi P., Islami F., Anandasabapathy S., Freedman N.D., Kamangar F. (2014). Gastric cancer: Descriptive epidemiology, risk factors, screening, and prevention. Cancer Epidemiol. Biomarkers Prev..

[4] Gullo I., Carneiro F., Oliveira C., Almeida G.M. (2018). Heterogeneity in Gastric Cancer: From Pure Morphology to Molecular Classifications. Pathobiology.

[5] Bang Y.J., Van Cutsem E., Feyereislova A., Chung H.C., Shen L., Sawaki A., Lordick F., Ohtsu A., Omuro Y., Satoh T. (2010). Trastuzumab in combination with chemotherapy versus chemotherapy alone for treatment of HER2-positive advanced gastric or gastro-oesophageal junction cancer (ToGA): A phase 3, open-label, randomised controlled trial. Lancet.

[6] Fuchs C.S., Tomasek J., Yong C.J., Dumitru F., Passalacqua R., Goswami C., Safran H., Dos Santos L.V., Aprile G., Ferry D.R. (2014). Ramucirumab monotherapy for previously treated advanced gastric or gastro-oesophageal junction adenocarcinoma (REGARD): An international, randomised, multicentre, placebo-controlled, phase 3 trial. Lancet.

[7] Wilke H., Muro K., Van Cutsem E., Oh S.C., Bodoky G., Shimada Y., Hironaka S., Sugimoto N., Lipatov O., Kim T.Y. (2014). Ramucirumab plus paclitaxel versus placebo plus paclitaxel in patients with previously treated advanced gastric or gastro-oesophageal junction adenocarcinoma (RAINBOW): A double-blind, randomised phase 3 trial. Lancet Oncol..

[8] Ohtsu A., Shah M.A., Van Cutsem E., Rha S.Y., Sawaki A., Park S.R., Lim H.Y., Yamada Y., Wu J., Langer B. (2011). Bevacizumab in combination with chemotherapy as first-line therapy in advanced gastric cancer: A randomized, double-blind, placebo-controlled phase III study. J. Clin. Oncol..

[9] Waddell T., Chau I., Cunningham D., Gonzalez D., Okines A.F., Okines C., Wotherspoon A., Saffery C., Middleton G., Wadsley J. (2013). Epirubicin, oxaliplatin, and capecitabine with or without panitumumab for patients with previously untreated advanced oesophagogastric cancer (REAL3): A randomised, open-label phase 3 trial. Lancet Oncol..

[10] Catenacci D.V.T., Tebbutt N.C., Davidenko I., Murad A.M., Al-Batran S.E., Ilson D.H., Tjulandin S., Gotovkin E., Karaszewska B., Bondarenko I. (2017). Rilotumumab plus epirubicin, cisplatin, and capecitabine as first-line therapy in advanced MET-positive gastric or gastro-oesophageal junction cancer (RILOMET-1): A randomised, double-blind, placebo-controlled, phase 3 trial. Lancet Oncol..

[11] Shah M.A., Cho J.Y., Tan I.B., Tebbutt N.C., Yen C.J., Kang A., Shames D.S., Bu L., Kang Y.K. (2016). A Randomized Phase II Study of FOLFOX With or Without the MET Inhibitor Onartuzumab in Advanced Adenocarcinoma of the Stomach and Gastroesophageal Junction. Oncologist.

[12] Van Cutsem E., Bang Y.J., Mansoor W., Petty R.D., Chao Y., Cunningham D., Ferry D.R., Smith N.R., Frewer P., Ratnayake J. (2017). A randomized, open-label study of the efficacy and safety of AZD4547 monotherapy versus paclitaxel for the treatment of advanced gastric adenocarcinoma with FGFR2 polysomy or gene amplification. Ann. Oncol..

[13] Turner N., Grose R. (2010). Fibroblast growth factor signalling: From development to cancer. Nat. Rev. Cancer.

[14] Babina I.S., Turner N.C. (2017). Advances and challenges in targeting FGFR signalling in cancer. Nat. Rev. Cancer.

[15] Nagatsuma A.K., Aizawa M., Kuwata T., Doi T., Ohtsu A., Fujii H., Ochiai A. (2015). Expression profiles of HER2, EGFR, MET and FGFR2 in a large cohort of patients with gastric adenocarcinoma. Gastric Cancer.

[16] Murase H., Inokuchi M., Takagi Y., Kato K., Kojima K., Sugihara K. (2014). Prognostic significance of the co-overexpression of fibroblast growth factor receptors 1, 2 and 4 in gastric cancer. Mol. Clin. Oncol..

[17] Su X., Zhan P., Gavine P.R., Morgan S., Womack C., Ni X., Shen D., Bang Y.J., Im S.A., Ho Kim W. (2014). FGFR2 amplification has prognostic significance in gastric cancer: Results from a large international multicentre study. Br. J. Cancer.

[18] Cancer Genome Atlas Research N. (2014). Comprehensive molecular characterization of gastric adenocarcinoma. Nature.

[19] Tokunaga R., Imamura Y., Nakamura K., Ishimoto T., Nakagawa S., Miyake K., Nakaji Y., Tsuda Y., Iwatsuki M., Baba Y. (2016). Fibroblast growth factor receptor 2 expression, but not its genetic amplification, is associated with tumor growth and worse survival in esophagogastric junction adenocarcinoma. Oncotarget.

[20] Tabernero J., Bahleda R., Dienstmann R., Infante J.R., Mita A., Italiano A., Calvo E., Moreno V., Adamo B., Gazzah A. (2015). Phase I Dose-Escalation Study of JNJ-42756493, an Oral Pan-Fibroblast Growth Factor Receptor Inhibitor, in Patients With Advanced Solid Tumors. J. Clin. Oncol..

[21] Ishii H., Hattori Y., Itoh H., Kishi T., Yoshida T., Sakamoto H., Oh H., Yoshida S., Sugimura T., Terada M. (1994). Preferential expression of the third immunoglobulin-like domain of K-sam product provides keratinocyte growth factor-dependent growth in carcinoma cell lines. Cancer Res..

[22] Ornitz D.M., Xu J., Colvin J.S., McEwen D.G., MacArthur C.A., Coulier F., Gao G., Goldfarb M. (1996). Receptor specificity of the fibroblast growth factor family. J. Biol. Chem..

[23] Johnson D.E., Lu J., Chen H., Werner S., Williams L.T. (1991). The human fibroblast growth factor receptor genes: A common structural arrangement underlies the mechanisms for generating receptor forms that differ in their third immunoglobulin domain. Mol. Cell Biol..

[24] Yayon A., Zimmer Y., Shen G.H., Avivi A., Yarden Y., Givol D. (1992). A confined variable region confers ligand specificity on fibroblast growth factor receptors: Implications for the origin of the immunoglobulin fold. EMBO J..

[25] Yoshino M., Ishiwata T., Watanabe M., Matsunobu T., Komine O., Ono Y., Yamamoto T., Fujii T., Matsumoto K., Tokunaga A. (2007). Expression and roles of keratinocyte growth factor and its receptor in esophageal cancer cells. Int. J. Oncol..

[26] Ishiwata T., Friess H., Buchler M.W., Lopez M.E., Korc M. (1998). Characterization of keratinocyte growth factor and receptor expression in human pancreatic cancer. Am. J. Pathol..

[27] Kurban G., Ishiwata T., Kudo M., Yokoyama M., Sugisaki Y., Naito Z. (2004). Expression of keratinocyte growth factor receptor (KGFR/FGFR2 IIIb) in human uterine cervical cancer. Oncol. Rep..

[28] Yamayoshi T., Nagayasu T., Matsumoto K., Abo T., Hishikawa Y., Koji T. (2004). Expression of keratinocyte growth factor/fibroblast growth factor-7 and its receptor in human lung cancer: Correlation with tumour proliferative activity and patient prognosis. J. Pathol..

[29] Cho K., Ishiwata T., Uchida E., Nakazawa N., Korc M., Naito Z., Tajiri T. (2007). Enhanced expression of keratinocyte growth factor and its receptor correlates with venous invasion in pancreatic cancer. Am. J. Pathol..

[30] De Diez Medina S.G., Chopin D., El Marjou A., Delouvee A., LaRochelle W.J., Hoznek A., Abbou C., Aaronson S.A., Thiery J.P., Radvanyi F. (1997). Decreased expression of keratinocyte growth factor receptor in a subset of human transitional cell bladder carcinomas. Oncogene.

[31] Shoji K., Teishima J., Hayashi T., Ohara S., McKeehan W.L., Matsubara A. (2014). Restoration of fibroblast growth factor receptor 2IIIb enhances the chemosensitivity of human prostate cancer cells. Oncol. Rep..

[32] Naimi B., Latil A., Fournier G., Mangin P., Cussenot O., Berthon P. (2002). Down-regulation of (IIIb) and (IIIc) isoforms of fibroblast growth factor receptor 2 (FGFR2) is associated with malignant progression in human prostate. Prostate.

[33] Zhang Y., Wang H., Toratani S., Sato J.D., Kan M., McKeehan W.L., Okamoto T. (2001). Growth inhibition by keratinocyte growth factor receptor of human salivary adenocarcinoma cells through induction of differentiation and apoptosis. Proc. Natl. Acad. Sci. USA.

[34] Matsubara A., Kan M., Feng S., McKeehan W.L. (1998). Inhibition of growth of malignant rat prostate tumor cells by restoration of fibroblast growth factor receptor 2. Cancer Res..

[35] Ricol D., Cappellen D., El Marjou A., Gil-Diez-de-Medina S., Girault J.M., Yoshida T., Ferry G., Tucker G., Poupon M.F., Chopin D. (1999). Tumour suppressive properties of fibroblast growth factor receptor 2-IIIb in human bladder cancer. Oncogene.

[36] Thiery J.P., Sleeman J.P. (2006). Complex networks orchestrate epithelial-mesenchymal transitions. Nat. Rev. Mol. Cell Biol..

[37] Warzecha C.C., Carstens R.P. (2012). Complex changes in alternative pre-mRNA splicing play a central role in the epithelial-to-mesenchymal transition (EMT). Semin. Cancer Biol..

[38] Carstens R.P., Eaton J.V., Krigman H.R., Walther P.J., Garcia-Blanco M.A. (1997). Alternative splicing of fibroblast growth factor receptor 2 (FGF-R2) in human prostate cancer. Oncogene.

[39] Zhao Q., Caballero O.L., Davis I.D., Jonasch E., Tamboli P., Yung W.K., Weinstein J.N., Strausberg R.L., Yao J. (2013). Tumor-specific isoform switch of the fibroblast growth factor receptor 2 underlies the mesenchymal and malignant phenotypes of clear cell renal cell carcinomas. Clin. Cancer Res..

[40] Ahn S., Lee J., Hong M., Kim S.T., Park S.H., Choi M.G., Lee J.H., Sohn T.S., Bae J.M., Kim S. (2016). FGFR2 in gastric cancer: Protein overexpression predicts gene amplification and high H-index predicts poor survival. Mod. Pathol..

[41] Han N., Kim M.A., Lee H.S., Kim W.H. (2015). Evaluation of Fibroblast Growth Factor Receptor 2 Expression, Heterogeneity and Clinical Significance in Gastric Cancer. Pathobiology.

[42] Catenacci D.V., Tesfaye A., Tejani M., Cheung E., Eisenberg P., Scott A.J., Eng C., Hnatyszyn J., Marina N., Powers J. (2019). Bemarituzumab with modified FOLFOX6 for advanced FGFR2-positive gastroesophageal cancer: FIGHT Phase III study design. Future Oncol..

[43] Park S., Kim J.H., Jang J.H. (2007). Aberrant hypermethylation of the FGFR2 gene in human gastric cancer cell lines. Biochem. Biophys Res. Commun..

[44] Warzecha C.C., Shen S., Xing Y., Carstens R.P. (2009). The epithelial splicing factors ESRP1 and ESRP2 positively and negatively regulate diverse types of alternative splicing events. RNA Biol..

[45] Warzecha C.C., Jiang P., Amirikian K., Dittmar K.A., Lu H., Shen S., Guo W., Xing Y., Carstens R.P. (2010). An ESRP-regulated splicing programme is abrogated during the epithelial-mesenchymal transition. EMBO J..

[46] Wang X., Liu Y., Shao D., Qian Z., Dong Z., Sun Y., Xing X., Cheng X., Du H., Hu Y. (2016). Recurrent amplification of MYC and TNFRSF11B in 8q24 is associated with poor survival in patients with gastric cancer. Gastric. Cancer.

[47] Hayakawa A., Saitoh M., Miyazawa K. (2017). Dual Roles for Epithelial Splicing Regulatory Proteins 1 (ESRP1) and 2 (ESRP2) in Cancer Progression. Adv. Exp. Med. Biol..

[48] Ueda J., Matsuda Y., Yamahatsu K., Uchida E., Naito Z., Korc M., Ishiwata T. (2014). Epithelial splicing regulatory protein 1 is a favorable prognostic factor in pancreatic cancer that attenuates pancreatic metastases. Oncogene.

[49] Yae T., Tsuchihashi K., Ishimoto T., Motohara T., Yoshikawa M., Yoshida G.J., Wada T., Masuko T., Mogushi K., Tanaka H. (2012). Alternative splicing of CD44 mRNA by ESRP1 enhances lung colonization of metastatic cancer cell. Nat. Commun..

[50] Esteller M. (2008). Epigenetics in cancer. N. Engl. J. Med..

[51] Deaton A.M., Bird A. (2011). CpG islands and the regulation of transcription. Genes Dev..

[52] Warzecha C.C., Sato T.K., Nabet B., Hogenesch J.B., Carstens R.P. (2009). ESRP1 and ESRP2 are epithelial cell-type-specific regulators of FGFR2 splicing. Mol. Cell.

[53] Toyokawa T., Yashiro M., Hirakawa K. (2009). Co-expression of keratinocyte growth factor and K-sam is an independent prognostic factor in gastric carcinoma. Oncol. Rep..

[54] Matsunobu T., Ishiwata T., Yoshino M., Watanabe M., Kudo M., Matsumoto K., Tokunaga A., Tajiri T., Naito Z. (2006). Expression of keratinocyte growth factor receptor correlates with expansive growth and early stage of gastric cancer. Int. J. Oncol..

[55] Lei Z., Tan I.B., Das K., Deng N., Zouridis H., Pattison S., Chua C., Feng Z., Guan Y.K., Ooi C.H. (2013). Identification of molecular subtypes of gastric cancer with different responses to PI3-kinase inhibitors and 5-fluorouracil. Gastroenterology.

[56] Fagoonee S., Bearzi C., Di Cunto F., Clohessy J.G., Rizzi R., Reschke M., Tolosano E., Provero P., Pandolfi P.P., Silengo L. (2013). The RNA binding protein ESRP1 fine-tunes the expression of pluripotency-related factors in mouse embryonic stem cells. PLoS ONE.

[57] Kwon O.H., Park J.L., Kim M., Kim J.H., Lee H.C., Kim H.J., Noh S.M., Song K.S., Yoo H.S., Paik S.G. (2011). Aberrant up-regulation of LAMB3 and LAMC2 by promoter demethylation in gastric cancer. Biochem. Biophys. Res. Commun..

[58] GDC Data Portal. https://portal.gdc.cancer.gov/.

[59] Laddha S.V., Ganesan S., Chan C.S., White E. (2014). Mutational landscape of the essential autophagy gene BECN1 in human cancers. Mol. Cancer Res..

[60] Mermel C.H., Schumacher S.E., Hill B., Meyerson M.L., Beroukhim R., Getz G. (2011). GISTIC2.0 facilitates sensitive and confident localization of the targets of focal somatic copy-number alteration in human cancers. Genome Biol..

[61] Wang L., Sun J., Wu H., Liu S., Wang J., Wu B., Huang S., Li N., Wang J., Zhang X. (2012). Systematic assessment of reduced representation bisulfite sequencing to human blood samples: A promising method for large-sample-scale epigenomic studies. J. Biotechnol..

[62] Haeussler M., Zweig A.S., Tyner C., Speir M.L., Rosenbloom K.R., Raney B.J., Lee C.M., Lee B.T., Hinrichs A.S., Gonzalez J.N. (2019). The UCSC Genome Browser database: 2019 update. Nucleic Acids Res..

[63] (2014). R: A Language and Environment for Statistical Computing.

[64] Wickham H. (2016). ggplot2: Elegant Graphics for Data Analysis.

