# Supplementary material for: Integrated Analysis of Structural Variation and RNA Expression of FGFR2 and Its Splicing Modulator ESRP1 Highlight the ESRP1amp-FGFR2norm-FGFR2-IIIchigh Axis in Diffuse Gastric Cancer
Source: Cancers (Basel). 2019 Dec 25;12(1):70. doi: 10.3390/cancers12010070 (PMC7017189; doi:10.3390/cancers12010070)
Supplement: Supplementary file 1 [file cancers-12-00070-s001.pdf]

Supplementary Materials

Integrated Analysis of Structural Variation and RNA Expression of FGFR2 and Its Splicing Modulator ESRP1 Highlight the *ESRP1*^{amp}-*FGFR2*^{norm}-*FGFR2-IIIc*^{high} Axis in Diffuse Gastric Cancer

Sara Pinto Teles, Patrícia Oliveira, Marta Ferreira, Joana Carvalho, Pedro Ferreira and Carla Oliveira

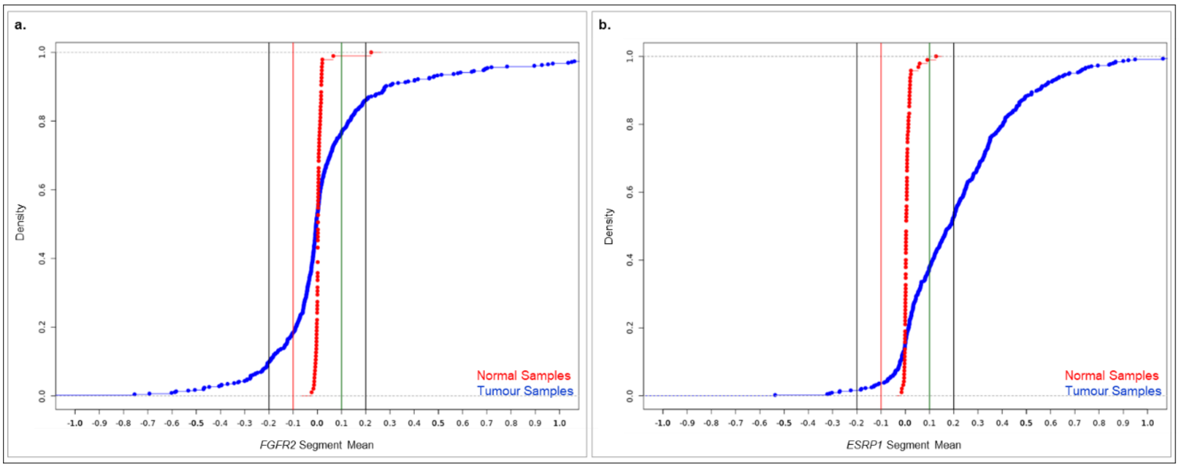

Figure S1. Distribution of gene segment mean values in cohort #1 dataset #1 and dataset #2 samples. (**a**) FGFR2. (**b**) ESRP1.

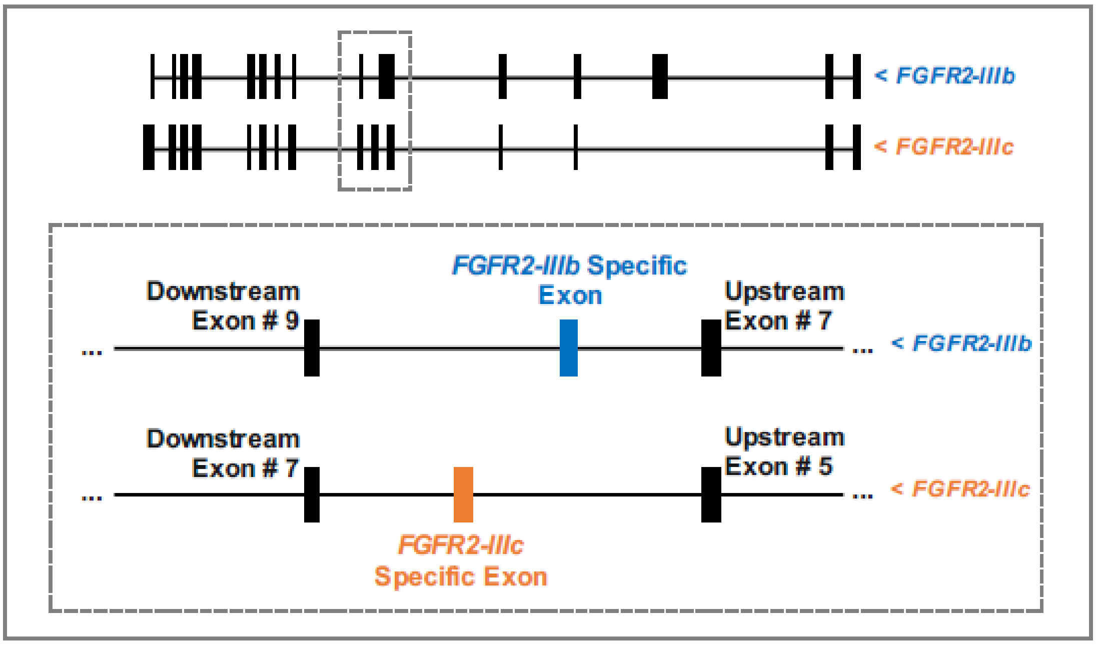

Figure S2. Representation of the alternative splicing underlying the two FGFR2 isoforms studied: FGFR2-IIIb and FGFR2-IIIc. In the dashed rectangle, a zoom in of the exons studied in more detail: FGFR2-IIIb specific exon, FGFR2-IIIc specific exon and the corresponding downstream and upstream exons, which correspond to exons 7 and 9 in the canonical FGFR2 transcript.

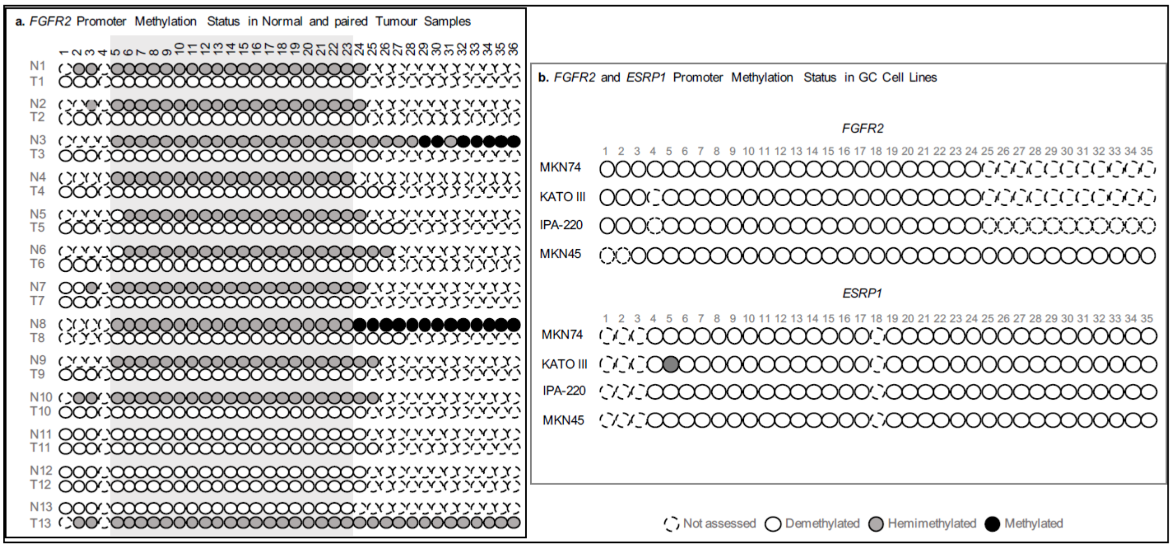

Figure S3. Schematic representation of FGFR2 and ESRP1 promoter regions evaluated by Bisulfite Sanger sequencing. (a) FGFR2 promoter proxy region evaluated by Bisulfite Sanger sequencing in 13 normal/tumour pairs from cohort #2. Circles stand for CpG sites coloured according to methylation status: white circles for demethylated CpG sites, grey circles for hemimethylated CpG sites, black circles for methylated CpG sites. Dashed circles for CpG sites not assessed. (b) Schematic representation of FGFR2 and ESRP1 promoter methylation status evaluated by Bisulfite Sanger sequencing of parentalMKN74, KATO III and IPA-220 and MKN45 GC cell lines.

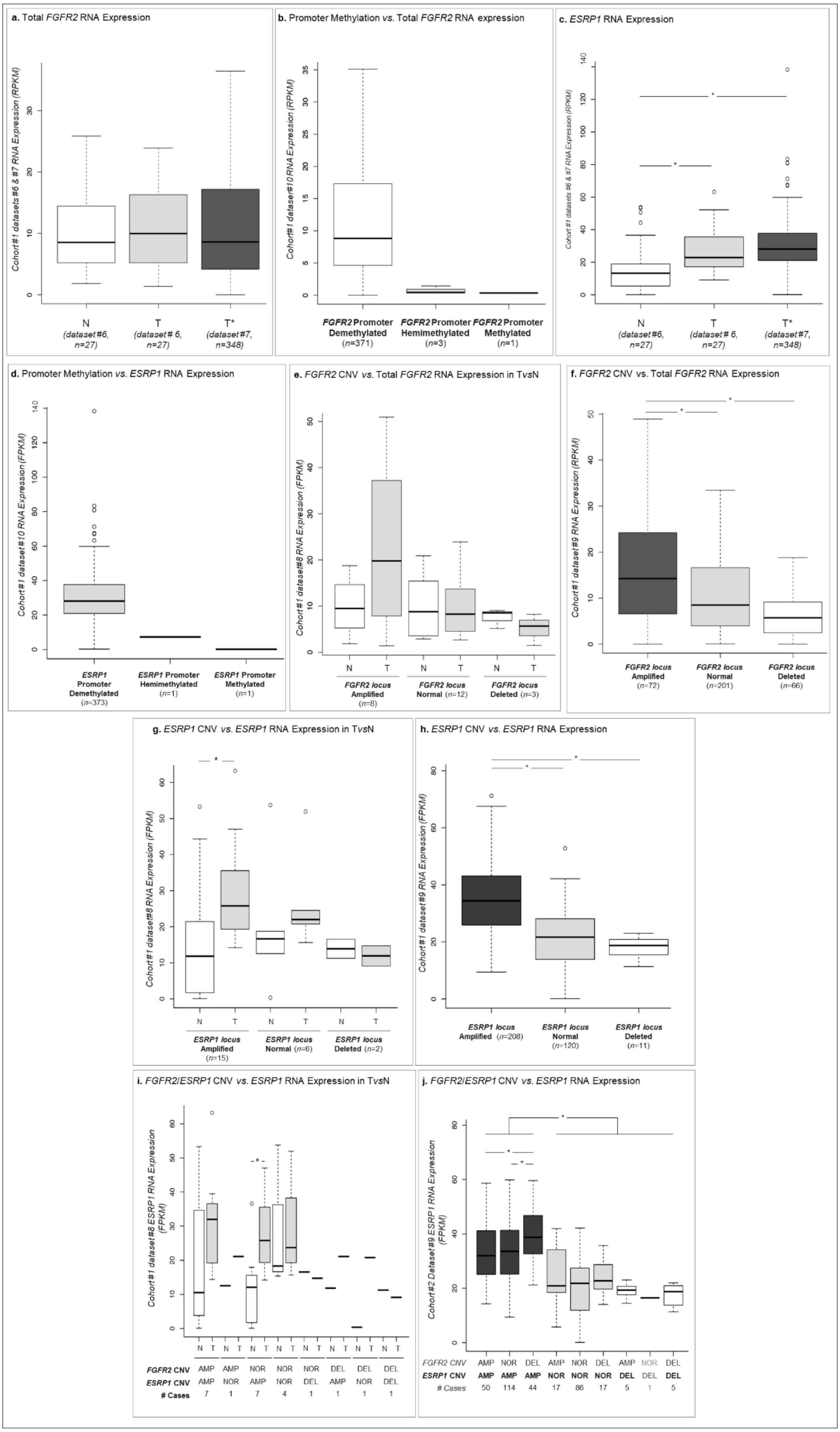

Figure S4. Total FGFR2 and ESRP1 RNA expression, CNV and promoter methylation status. (**a**) Total *FGFR2* RNA expression in TvsN and unpaired tumour samples from cohort #1 datasets #6 and #7. (**b**)

Total *FGFR2* RNA expression in tumours separated according to *FGFR2* promoter status (cohort #1 dataset #10). (**c**) Same as (**a**) for *ESRP1*. (**d**) Same as (**b**) for *ESRP1*. (**e**) Total *FGFR2* RNA expression in TvsN separated according to *FGFR2* CN status (cohort #1 dataset #8). (**f**) Total *FGFR2* RNA expression in tumours separated according to *FGFR2* CN status (cohort #1 dataset #9). (**g**) Same as (**e**) for *ESRP1***.** (**h**) Same as (**f**) for *ESRP1*. (**i**) *ESRP1* RNA expression in TvsN separated according to *FGFR2* and *ESRP1* CN status (cohort #1 dataset #8). All 9 possible combinations for amplified (AMP), normal (NOR) and deleted (DEL) were detected and are described in the X-axis along with the number of cases observed. Not all outliers are displayed. (**j**) *ESRP1* RNA expression in unpaired tumours separated according to *FGFR2* and *ESRP1* CN status (cohort #1 dataset #9). Asterisks correspond to *p*-value < 0.05.

A.

			FGFR2 CN								
			AMP			NOR			DEL		
		ESRP1 CN	AMP	NOR	DEL	AMP	NOR	DEL	AMP	NOR	DEL
FGFR2	AMP	AMP		*ns*	*ns*	1.61E-03	1.09E-03	*ns*	3.75E-05	2.10E-03	*ns*
		NORM			*ns*	1.16E-02	1.12E-02	*ns*	1.62E-04	3.05E-03	*ns*
		DEL				*ns*	*ns*	*ns*	*ns*	*ns*	*ns*
	NOR	AMP					*ns*	*ns*	4.41E-02	*ns*	*ns*
		NORM						*ns*	*ns*	*ns*	*ns*
		DEL							*ns*	*ns*	*ns*
	DEL	AMP								*ns*	*ns*
		NORM									*ns*
		DEL									

B.

			FGFR2 CN								
			AMP			NOR			DEL		
		ESRP1 CN	AMP	NOR	DEL	AMP	NOR	DEL	AMP	NOR	DEL
FGFR2	AMP	AMP		*ns*	*ns*	*ns*	*ns*	*ns*	5.29E-03	*ns*	*ns*
		NORM			*ns*	3.70E-02	*ns*	*ns*	2.94E-04	3.41E-02	*ns*
		DEL				*ns*	*ns*	*ns*	*ns*	*ns*	*ns*
	NOR	AMP					2.34E-02	*ns*	2.95E-02	*ns*	*ns*
		NORM						*ns*	1.33E-04	3.80E-02	*ns*
		DEL							*ns*	*ns*	*ns*
	DEL	AMP								*ns*	*ns*
		NORM									*ns*
		DEL									

C.

			FGFR2 CN								
			AMP			NOR			DEL		
		ESRP1 CN	AMP	NOR	DEL	AMP	NOR	DEL	AMP	NOR	DEL
FGFR2	AMP	AMP		1.81E-03	2.01E-03	*ns*	4.43E-09	*ns*	1.49E-02	8.51E-04	1.10E-03
		NORM			*ns*	1.20E-03	*ns*	*ns*	1.46E-05	*ns*	*ns*
		DEL				2.82E-03	*ns*	*ns*	4.41E-05	*ns*	*ns*
	NOR	AMP					2.59E-12	*ns*	4.92E-03	7.62E-04	1.58E-03
		NORM						*ns*	2.33E-13	*ns*	*ns*
		DEL							*ns*	*ns*	*ns*
	DEL	AMP								1.38E-07	3.04E-05
		NORM									*ns*
		DEL									

Figure S5. Significance matrices for RNA expression of *FGFR2-IIIb*, *FGFR2-IIIc* and *ESRP1* in cohort #1 dataset #9 GC cases separated and compared according to *FGFR2* and *ESRP1* CN status. (**a**) *FGFR2-IIIb* RNA expression. (**b**) *FGFR2-IIIc* RNA expression. (**c**) *ESRP1* expression. NS stands for $p>0.05$.

a.

Log Rank Test (*p*-values)	Diffuse & Above Normal Median	Diffuse & Below Normal Median	Intestinal & Above Normal Median	Intestinal & Below Normal Median	Mixed & Above Normal Median	Mixed & Below Normal Median
Diffuse & Above Normal Median		3,40E-02	*ns*	1,60E-02	*ns*	*ns*
Diffuse & Below Normal Median			*ns*	*ns*	5,00E-02	4,00E-03
Intestinal & Above Normal Median				*ns*	*ns*	1,50E-02
Intestinal & Below Normal Median					*ns*	2,00E-03
Mixed & Above Normal Median						*ns*
Mixed & Below Normal Median						

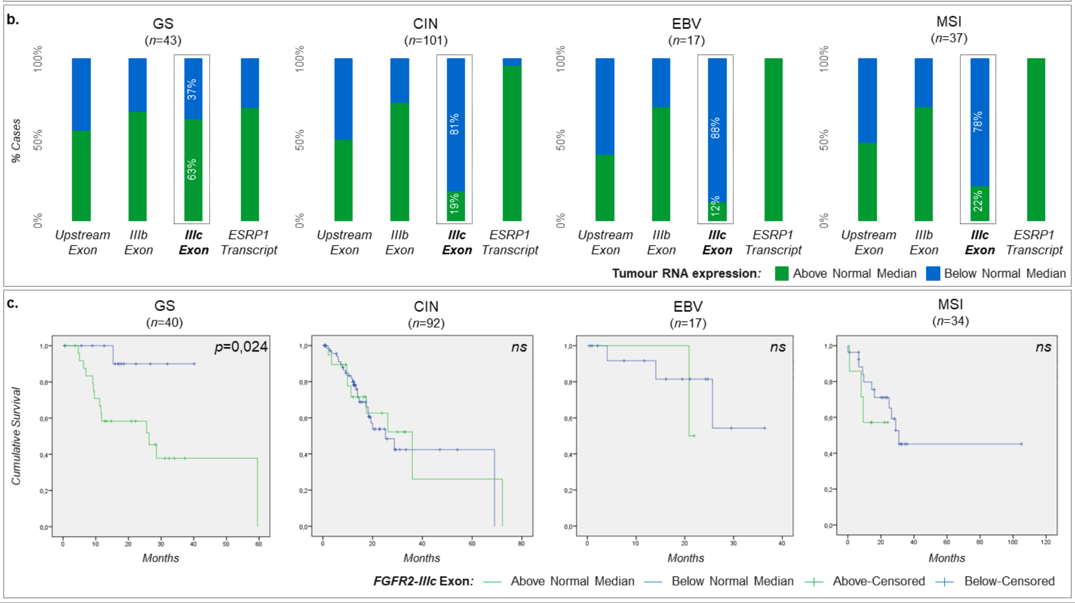

Figure S6. Correlation between *FGFR2-IIIc* expression and clinico-pathological features. (**a**) Log rank *p*-values obtained for the comparison between cumulative survival for GC (cohort #1 dataset #11) separated according to Lauren classification, represented in Figure 4c. (**b**) Percentage of GC divided according to TCGA molecular classification (Genomically Stable, GS; Chromosomal Instable, CIN; Epstein-Barr Virus positive, EBV and; Microsatellite instable, MSI) displaying RNA expression of *FGFR2* upstream and downstream exons, *FGFR2-IIIb* or *FGFR2-IIIc* specific exons above (green) or below (blue) the normal stomach median expression. (**c**) Kaplan-Meier plots for GS. CIN, EBV and MSI GC separated according to *FGFR2-IIIc* specific exon expression (above/below the median of normal stomach).

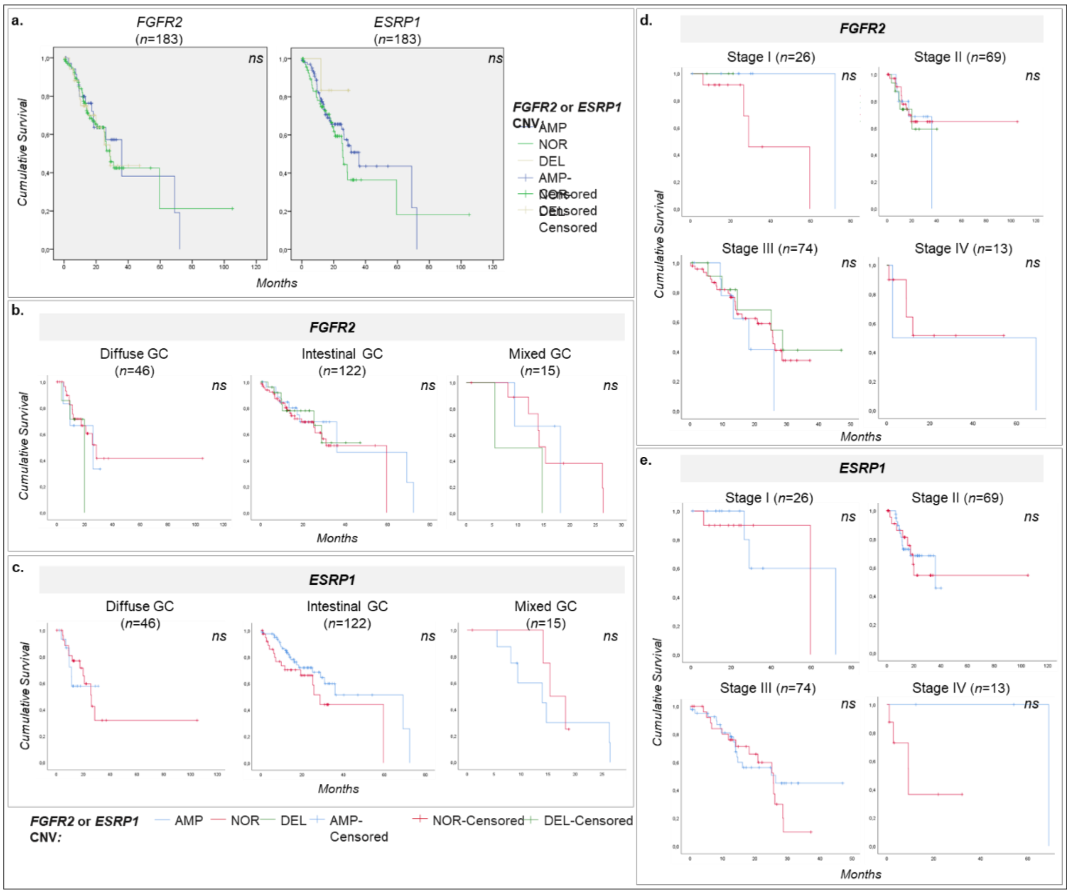

Figure S7. Correlation between *FGFR2* and *ESRP1* CN and clinico-pathological features. (**a**) Kaplan-Meier plots for cohort #1 dataset #11 GC separated according to *FGFR2* or *ESRP1* CN status: "AMP" for copy number amplification; "NOR" for normal copy number and; "DEL" for copy number deletion. (**b**) Kaplan-Meier plots for diffuse, intestinal or mixed GC separated according to *FGFR2* CN status. (**c**) Kaplan-Meier plots for diffuse, intestinal or mixed GC separated according to *ESRP1* CN status. No GC was detected with *ESRP1* copy number deletions. (**d**) Kaplan-Meier plots for stage I-IV GC separated according to *FGFR2* CN status. (**e**) Kaplan-Meier plots for stage I–IV GC separated according to *ESRP1* CN status.
